# Draft genome sequences of non-endemic *Mycobacterium tuberculosis* Indo-Oceanic and West African lineages recovered in Panama

**DOI:** 10.1128/mra.00760-26

**Published:** 2026-09-17

**Authors:** Juan C. Domínguez González, Joanna E. Rivas Ramos, Prudencio González, Pedro Del Cid, Paul R. Johnston, Samantha Rosas

**Affiliations:** 1 Mycobacteriology Section, Central Public Health Reference Laboratory, Conmemorative Gorgas Institute (ICGES) 199319 , Panama, Panama; 2 International Center for Life and Health Sciences Research (CIVSAL), Faculty of Medicine and Faculty of Engineering, Universidad Latina de Panamá 125801, Panama, Panama; 3 School of Medicine, University of St Andrews 7486, St Andrews, Scotland, United Kingdom; Queens College Department of Biology, Queens, New York, USA

**Keywords:** *Mycobacterium tuberculosis*, tuberculosis, DNA sequencing, bioinformatics, multidrug resistance

## Abstract

We report the draft genome sequences of three *Mycobacterium tuberculosis* isolates recovered from Panamanian residents belonging to the Indo-Oceanic (Lineage 1) and West African (Lineage 5) lineages. These isolates exhibit various drug-resistance profiles, including rifampicin-resistant and multidrug-resistant phenotypes. This highlights the changing molecular epidemiology of tuberculosis in Central America.

## ANNOUNCEMENT

*Mycobacterium tuberculosis* (*MTB*) remains the world’s leading cause of death from a single infectious agent (1, 2). Although *M. tuberculosis complex* (MTBC) shows strong phylogeographic structure, Panama’s role as a transit hub may facilitate the introduction and local establishment of lineages typically restricted to regions outside the Western Hemisphere (3–6). Genomic surveillance is needed to distinguish isolated importations from emerging local transmission.

Here, we report draft genome sequences of three *MTB* clinical isolates recovered from Panamanian residents: PAN-049, PAN-056, and PAN-043. Phylogenetic analysis identified two isolates belonging to the Indo-Oceanic lineage (Lineage 1, Panamá Province) and one to the West African lineage (Lineage 5, Chiriquí Province).

Isolates were recovered from sputum of pulmonary TB patients under protocol N°115/CBI/ICGES/25 approved by the Comité de Bioética de la Investigación del Instituto Conmemorativo Gorgas de Estudios de la Salud (CBI/ICGES). Samples were decontaminated using N-acetyl-L-cysteine-sodium hydroxide. Strains stored in 10% skim milk at −80°C were subcultured on solid Löwenstein–Jensen medium at 37°C ± 1°C for up to 4 weeks until confluent growth. Parallel liquid culture was performed using the BacT/ALERT MP system (bioMérieux) incubated at 37°C ± 1°C for up to 6 weeks. Confirmation of MTBC was determined using Line Probe Assays (GenoType MTBDRplus, Hain Lifescience). Genomic DNA was extracted using the QIAamp DNA Mini Kit (QIAGEN), and concentrations were determined using the Qubit dsDNA HS Assay Kit (Invitrogen). Sequencing was performed on the MiSeq platform (Illumina) using Nextera XT DNA Library Prep chemistry with paired-end chemistry (2 × 250 bp).

In total, 2,144,812 reads were obtained for PAN-049, 1,221,430 for PAN-056, and 1,557,206 for PAN-043, yielding average genome coverages of 64.34×, 36.64×, and 53.08×, respectively. Raw paired-end read quality was evaluated using FastQC v0.12.1 (3), which confirmed high per-base sequence quality and minimal adapter content. *De novo* assembly was performed using Unicycler v0.5.1 (4), and quality was evaluated using QUAST v5.3.0 (5). The final assembly lengths were 4,314,283 bp for PAN-049 (121 contigs; N50, 80,392 bp), 4,308,930 bp for PAN-056 (159 contigs; N50, 60,442 bp), and 4,314,986 bp for PAN-043 (149 contigs; N50, 64,724 bp), with a consistent GC content of 65% across all isolates. Genome annotation was performed using the National Center for Biotechnology Information (NCBI) Prokaryotic Genome Annotation Pipeline 2026-06-18.build8602, utilizing GeneMarkS-2+for structural gene prediction and CheckM (6–10). Default parameters were used in all software packages, except where otherwise noted.

**Fig 1:**
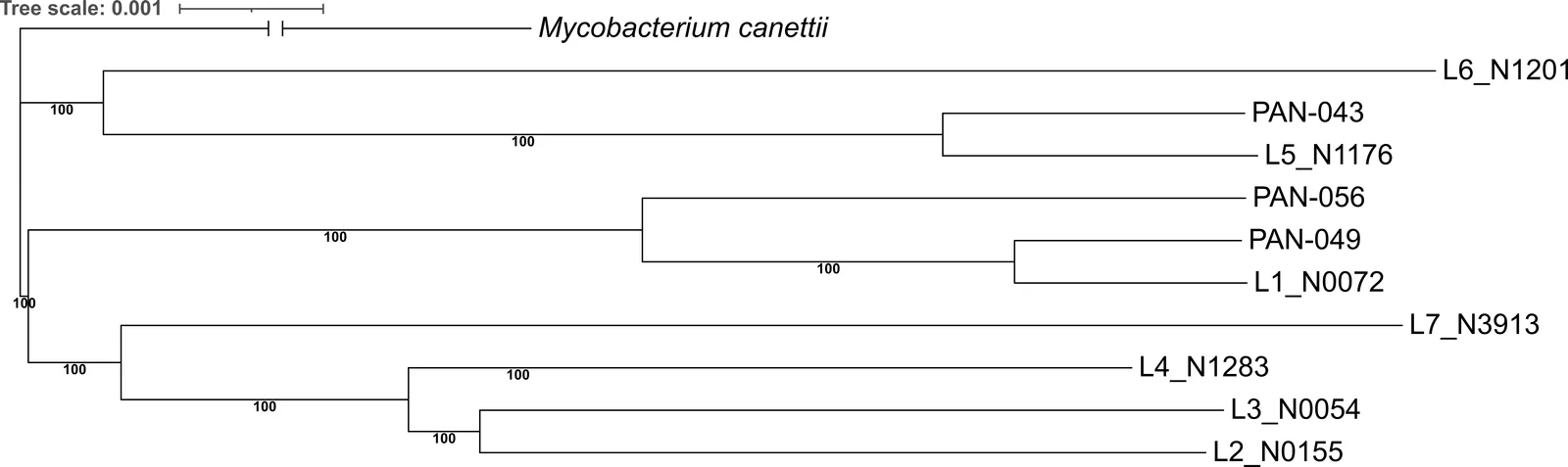
Core-genome maximum-likelihood phylogeny showing the evolutionary placement of Panamanian *Mycobacterium tuberculosis* complex isolates (PAN-043, PAN-049, and PAN-056) relative to Lineages 1–7 global reference strains, rooted on *Mycobacterium canettii*. Isolates PAN-049 and PAN-056 cluster within the Lineage 1 clade (L1_N0072) and PAN-043 clusters within the Lineage 5 clade (L5_N1176). The branch leading to *Mycobacterium canettii* has been visually truncated (indicated by the branch break symbol) to enable resolution of internal MTBC branching. Node values denote ultrafast bootstrap support percentages (100%). Scale bar represents 0.001 substitutions per site.

Lineage assignment and resistance profiling were performed by mapping reads to the *MTB* H37Rv (AL123456.3) using TB-profiler v6.7.0 (11). PAN-049 (1.1.2) and PAN-056 (1.2.1.2.1) were classified as RR-TB with mutations in the *rpoB* gene (*Gln432Leu* and *Ser450Leu,* respectively) (12, 13). PAN-043 was identified as Lineage 5.2 and was classified as MDR-TB (Fig. 1) with *inhA (*−*15C* > *T*) and *rpoB* (*Ser450Leu*) mutations, along with the *embB* (*Met306Val*) (14, 15). To confirm lineage assignments, core-genome single nucleotide polymorphisms (SNPs) were identified using Snippy v4.6.0, and a maximum-likelihood phylogeny was reconstructed using IQ-TREE v3.1.3 (16, 17) with 1,000 ultrafast bootstrap replicates, incorporating *Mycobacterium canettii* (SRR29477258) as an outgroup and global MTBC reference strains representing Lineages 1–7 (18, 19) (Fig. 1).

## Data Availability

The complete genomic sequences and data associated with the *Mycobacterium tuberculosis* strains analyzed in this study have been deposited in NCBI under PRJNA1476947. The Illumina read data for these isolates are available under the following Sequence Read Archive run accessions: SRR39088419 (PAN-049, GenBank assembly JCAOGZ000000000), SRR39088411 (PAN-056, GenBank assembly JCAOGY000000000), and SRR39088425 (PAN-043, GenBank assembly JCAOGX000000000).
